# Secondary immunoglobulin A nephropathy with gross hematuria leading to rapidly progressive glomerulonephritis following severe acute respiratory syndrome coronavirus 2 vaccination: a case report

**DOI:** 10.1186/s12882-023-03287-y

**Published:** 2023-08-08

**Authors:** Miyako Fukuda, Tomohiro Kaneko, Takahiro Kawai, Hiromasa Ishii, Akira Shimizu

**Affiliations:** 1 Department of Nephrology, Nippon Medical School Tama-Nagayama Hospital, Tama, Tokyo, Japan; 2 Department of Analytic Human Pathology, Nippon Medical School, Bunkyo-Ku, Tokyo, Japan

**Keywords:** COVID-19, SARS-CoV-2 vaccine, IgA nephropathy, Case report, Gross hematuria, Rapidly progressive glomerulonephritis, Secondary IgA, Cirrhosis

## Abstract

**Background:**

The outbreak of severe acute respiratory syndrome coronavirus 2 (SARS-CoV-2) infection has been followed by many reports of the development and relapse of autoimmune diseases associated with SARS-CoV-2 vaccination. Some of these reports have involved relapse or onset of immunoglobulin A (IgA) nephropathy following SARS-CoV-2 vaccination. Here, we report on a patient with IgA nephropathy who presented with gross hematuria and rapidly progressive glomerulonephritis following SARS-CoV-2 vaccination.

**Case presentation:**

A 63-year-old male patient with a history of habitual tonsillitis underwent bilateral tonsillectomy. He had a history of alcoholic cirrhosis of the liver and microscopic hematuria and proteinuria were indicated during a health checkup 2 years before hospital admission. He developed hematuria after the SARS-CoV-2 vaccination, which led to rapidly progressive glomerulonephritis, for which he was hospitalized. A renal biopsy led to the diagnosis of IgA nephropathy. Although pulse steroid therapy during his condition resulted in hepatic encephalopathy, three courses combined with mizoribine improved his renal function.

**Conclusion:**

SARS-CoV-2 mRNA vaccines activate T cells, which are involved in the pathophysiology of IgA nephropathy. Therefore, this case suggests that the exacerbation of IgA nephropathy by the vaccine favors the vasculitis aspect of the disease.

## Background

Immunoglobulin A (IgA) nephropathy is characterized by proliferative changes in glomerular mesangial cells and matrix and depositions primarily comprising IgA in the mesangial region. IgA nephropathy is the most frequent biopsy-proven primary glomerular disease; however, its geographic distribution varies widely. The frequency of IgA nephropathy is estimated to be 40%, 20%, and 2%–10% in East Asia, Europe, and North America, respectively [1, 2], and this is influenced by the different indications for renal biopsy in different countries and centers [3, 4]. Secondary IgA nephropathy results from conditions such as liver and connective tissue disease, malignancies, and infections [2, 5]. However, acute kidney injury with gross hematuria is reported to occur in ˂5% of all cases of IgA nephropathy [6].

There have been reports of new [7–12] or worsening of pre-existing cases of IgA nephropathy [8, 13–16] following the current severe acute respiratory syndrome coronavirus 2 (SARS-CoV-2) vaccinations. To the best of our knowledge, no reports of patients with IgA nephropathy secondary to cirrhosis, deteriorating and developing rapidly progressive glomerulonephritis (RPGN) following SARS-CoV-2 vaccination are available.

Here, we report on a patient with IgA nephropathy who presented with gross hematuria and RPGN following SARS-CoV-2 vaccination.

## Case presentation

A 63-year-old Japanese male patient presented with gross hematuria and RPGN following SARS-CoV-2 vaccination. The patient had a history of alcoholic cirrhosis of the liver, and a health checkup 2 years earlier indicated proteinuria (1 +) and microscopic hematuria (3 +) when his serum creatinine (Cr) and blood urea nitrogen (BUN) levels were 0.99 mg/dL and 9.4 mg/dL, respectively. He also had a history of habitual tonsillitis, for which he underwent a bilateral tonsillectomy when he was 12. He had no other relevant medical history and no family history of IgA nephropathy or other forms of kidney disease.

The patient manifested gross hematuria and general malaise a day after receiving his second dose of the SARS-CoV-2 vaccine manufactured by Pfizer. Urinalysis performed 15 days after vaccination revealed proteinuria (3 +) and occult blood (3 +); his serum Cr level was 4.07 mg/dL. Following examination at the Department of Nephrology 25 days after vaccination, he was admitted to the hospital because of RPGN. Although the patient had bilateral pitting pedal edema, no periorbital edema was observed. There was no cervical lymphadenopathy, pharyngeal erythema, or purpura on his legs. He was afebrile (with a body temperature of 36.4°C), and his blood pressure was 154/73 mmHg. Abdominal computed tomography revealed dull liver edges, irregular surface, and mild liver enlargement. The left and right kidneys were enlarged, measuring 127 mm and 120 mm in length, respectively.

Initial testing on admission (Table 1) revealed anemia and thrombocytopenia (hemoglobin, 10.9 g/dL; packed cell volume, 32.4%; and platelet count, 111,000/µL) and a white blood cell count of 4600/µL. The coagulation test (prothrombin time, 14.9 s; activated partial thromboplastin time, 36.0 s) and inflammation marker levels (C-reactive protein = 0.14 mg/dL) were normal. However, liver function tests revealed abnormal results (total protein, 7.7 g/dL; albumin, 3.2 g/dL; aspartate transaminase, 47 IU/L; alanine transaminase, 30 IU/L; lactate dehydrogenase, 351 IU/L; alkaline phosphatase, 94 IU/L; and gamma-glutamyl transpeptidase, 224 IU/L); renal function tests also revealed abnormal results (urea nitrogen, 29.3 mg/dL; Cr, 4.5 mg/dL), and the serum levels of IgG (2058 mg/dL) and IgA (1074 mg/dL) were high. Antibody test results (antinuclear antibodies, myeloperoxidase–antineutrophil cytoplasmic antibody [ANCA], PR3-ANCA, and anti-glomerular basement membrane antibodies) were all negative. The patient’s serum complements C3 (104 mg/dL) and C4 (11/5 mg/dL) levels were normal. However, tests for hepatitis B, hepatitis C, and human immunodeficiency viruses were negative. Urinalysis revealed abnormalities (proteins, 3 + ; occult blood, 3 + ; sediment red blood cells > 100 high-power field [HPF]; glomerular red blood cells, 50 − 99/HPF; hyaline casts > 100/whole field; urinary protein, 319 mg/dL; urinary Cr, 126.2 mg/dL; and urinary β2-MG, 3278 ug/L). The results of SARS-CoV-2 nucleic acid amplification testing using loop-mediated isothermal amplification were negative.

**Table 1:** Test findings at admission

Parameter	Value (reference range)
Hematology	
WBC (µ/L)	4600 (3300–8600)
Hb (mg/dL)	10.9 (11.6–14.8)
Plt (10^4^/µL)	11.1 (15.8–34.8)
Blood chemistry	
TP (g/dL)	7.7 (6.7–8.3)
Alb (g/dL)	3.2 (3.9–4.9)
BUN (mg/dL)	29.3 (8.0–20.0)
Cr (mg/dL)	4.5 (0.40–0.80)
eGFR (mL/min/1.73 m^2^)	11.4 (> 60)
AST (U/L)	47 (8–38)
ALT (U/L)	30 (4–44)
LDH (U/L)	351 (106–211)
FBS (mg/dL)	133 (70–110)
HbA1c (%)	5.7 (4.6–6.2)
TG (mg/dL)	113 (50–149)
LDL-C (mg/dL)	90 (70–139)
Immunology	
IgG (mg/dL)	2058 (820–1740)
IgA (mg/dL)	1074 (90–400)
IgM (mg/dL)	217 (52–270)
C3 (mg/dL)	104 (80–140)
C4 (mg/dL)	11.5 (11–34)
CH50 (mg/dL)	26 (30–45)
CRP (mg/dL)	0.14 (< 0.30)
Antinuclear antibody (IU/mL)	< 40 (< 40)
MPO-ANCA (U/mL)	< 0.5 (< 0.5)
PR3-ANCA (U/mL)	< 0.5 (< 0.5)
Anti-GBM antibody (IU/mL)	< 5.0 (< 5.0)
Urinalysis	
Occult blood	3 +
Dipstick protein	3 +
RBC (/HPF)	50–99 (< 5)
Protein urea (g/g creatinine)	2.59 (< 0.15)

*Alb* albumin, *ALT* alanine transaminase, *AST*, aspartate transaminase, *BUN* blood urea nitrogen, *Cr* creatinine, *CRP* C-reactive protein, *eGFR* estimated glomerular filtration rate, *FBS* fasting blood sugar, *GBM* glomerular basement membrane, *Hb* hemoglobin, *HbA1c* glycated hemoglobin, *HPF* high power field, *LDH* lactate dehydrogenase, *LDL-C* low-density lipoprotein cholesterol, *MPO-ANCA* myeloperoxidase–antineutrophil cytoplasmic antibody, *Plt* platelet, *RBC* red blood cell, *TG* triglyceride, *TP* total protein, *WBC* white blood cell

On day 6 of hospitalization, a renal biopsy was performed, and the resected specimen included 13–17 glomeruli.

**Fig. 1:**
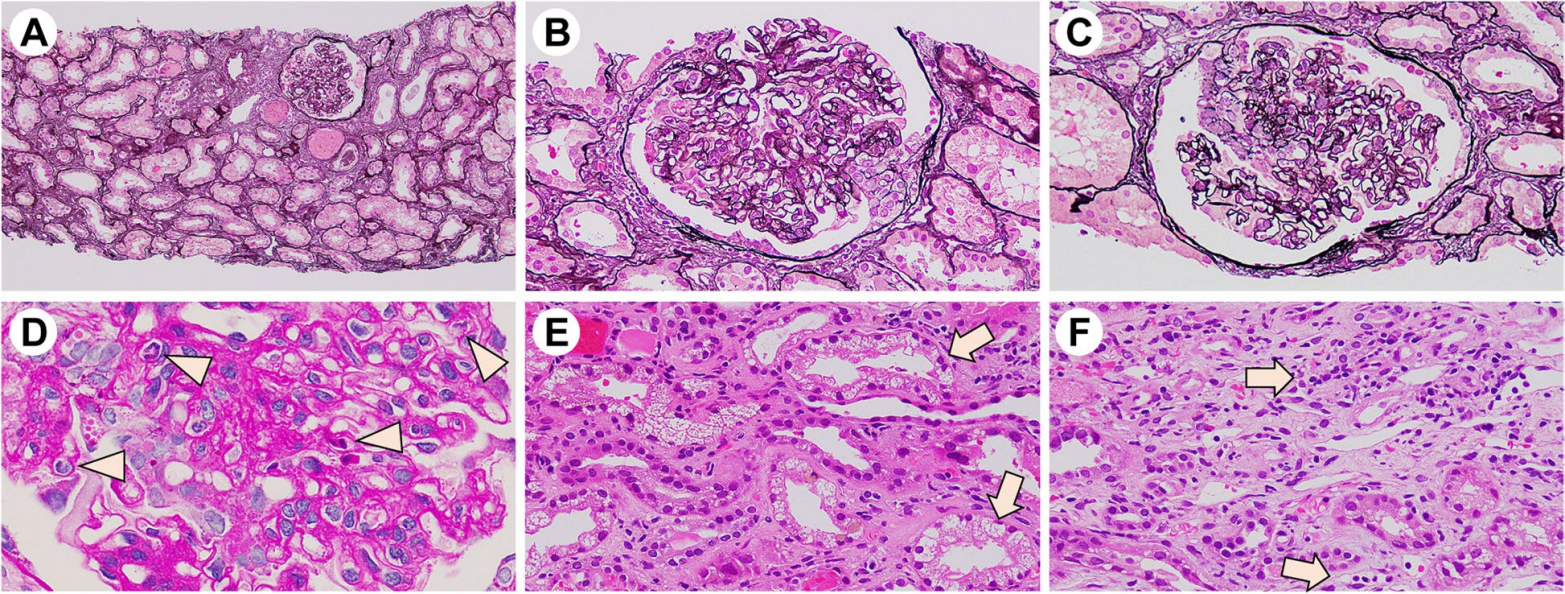
Renal biopsy light microscopy findings. **a** One glomerulus demonstrating proliferative lesions, erythrocyte casts in the distal tubules, and expansion of the interstitium (PAM stain; original magnification × 200). **b**, **c** Another glomerulus demonstrating proliferative lesions and the formation of a small cellular crescent in Bowman’s space (PAM stain; original magnification × 400). **d** Infiltration of neutrophils in loops (arrows) is observed in the glomeruli (PAS stain; original magnification × 400). **e** Renal proximal tubule epithelial cells demonstrating vacuolar degeneration (arrows), while the interstitium has expanded and demonstrates mild inflammatory cell infiltration. The distal renal tubules demonstrating erythrocyte casts (hematoxylin and eosin stain; original magnification × 400). **f** Inflammatory cell infiltration is observed in medullary capillaries (arrows), as well as in the surrounding interstitium; these findings reveal medullary capillaritis (hematoxylin and eosin stain; original magnification × 400). AM, Periodic acid-methenamine silver

Glomeruli showed inflammatory cell infiltration and cellular crescent formation. Renal proximal tubule epithelial cells showed vacuolar degeneration and dysfunction. Inflammatory cell infiltration was also observed in the cortical interstitium. Findings that are consistent with glomerular capillaritis were also observed. Additionally, cortical inflammatory cell infiltration and medullary capillaritis were observed, suggesting an enhanced cortical and medullary inflammatory response in addition to an enhanced glomerular inflammatory response (Fig. 1). Mesangial cell proliferation was detected in 35% of the cases, and no evidence of endocapillary hypercellularity, global glomerulosclerosis, segmental glomerulosclerosis, and tubular atrophy/interstitial fibrosis was observed. The examination revealed 2/17 cellular and 1/17 fibrocystic crescents. MEST-C scores were M0, E0, S0, T0, and C1. Medullary angiitis, which is usually present in ANCA, was absent.

**Fig. 2:**
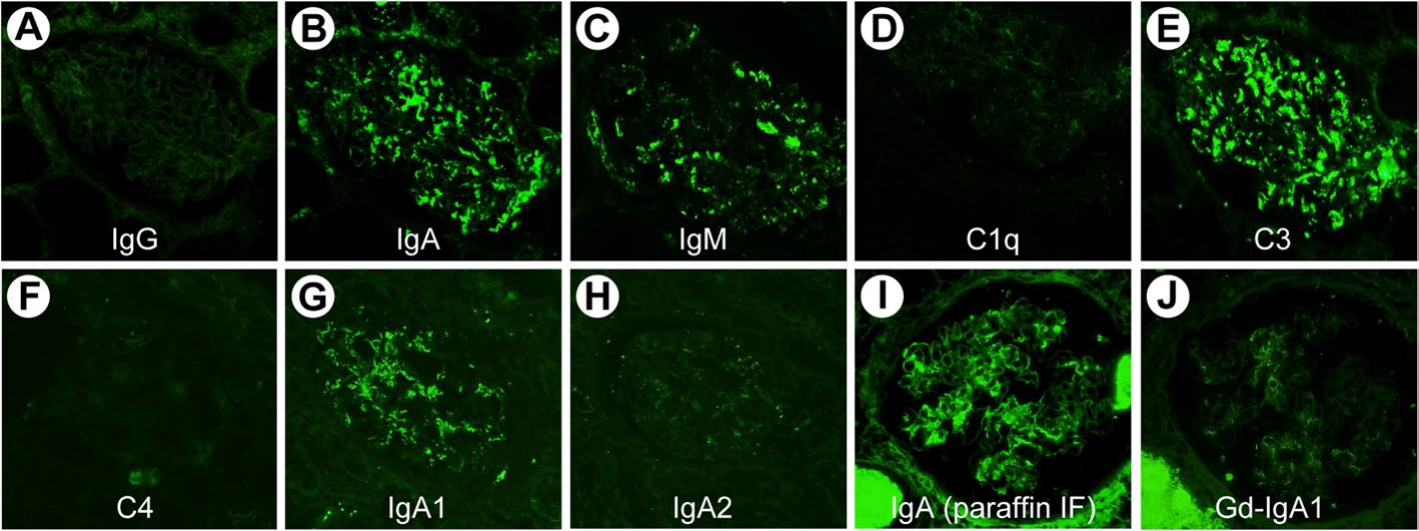
Immunofluorescence findings. Immunofluorescence reveals a mesangial pattern consisting primarily of IgA( +) and C3( +). IgM( ±) segmental mesangial findings are also observed. The IgA subclass IgA1 is dominant

Immunofluorescence revealed a mesangial pattern primarily comprising IgA( +) and C3( +). IgM( ±) segmental mesangial findings were also observed. The IgA subclass IgA1 was dominant, and mesangial granular findings were also observed in IgA on paraffin immunofluorescence. Although findings suggestive of galactose-deficient IgA1 were present, these were weak compared to IgA. These findings were consistent with those of secondary IgA (Fig. 2).

**Fig. 3:**
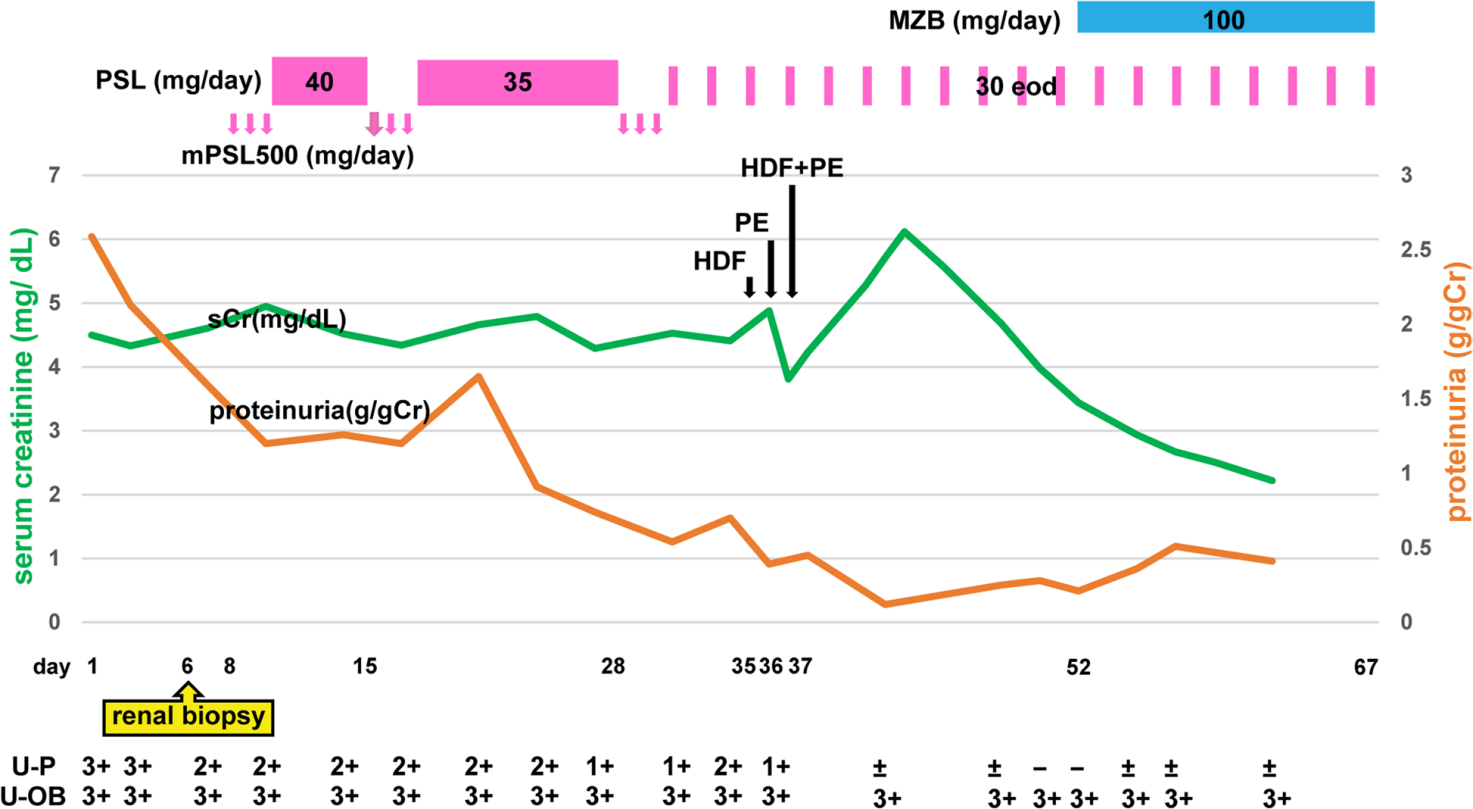
Clinical course. Abbreviations: eod, each other day; HDF, Hemodiafiltration; mPSL, methylprednisolone; MZR, mizoribine; PE, plasma exchange; PSL, prednisolone; sCr, serum creatinine; U-OB, urine occult blood; U-P, urine protein; U-OB, urine occult blood

The patient was diagnosed with IgA nephropathy and began receiving pulse steroid therapy (methylprednisolone, 500 mg/day for 3 days) on day 7 of hospitalization. On day 10, he began receiving oral prednisolone (PSL) at 40 mg/day. On day 15, he received two courses of pulse steroid therapy, and from day 19 onward, the dose of PSL was reduced to 35 mg/day. Before admission, the patient took olmesartan medoxomil but discontinued upon admission because of worsening renal function. Subsequently, we managed the patient’s blood pressure using a calcium channel blocker. We resumed losartan potassium on day 27 as the patient’s renal dysfunction peaked. The third course of pulse steroid therapy was initiated on day 28, and on day 34, his level of consciousness began to decline, and he became delirious. The serum levels of BUN, Cr, total bilirubin, and ammonia had increased to 102.9 mg/dL, 4.88 mg/dL, 4.1 mg/dL, and 181 µg/dL, respectively, suggesting acute liver failure associated with pulse steroid therapy. He was commenced on hemodiafiltration and plasma exchange on days 35 and 36, respectively. Hemodiafiltration was performed to remove inflammatory cytokines and small- to medium-molecular toxic substances and to promote awakening from hepatic encephalopathy. In toal, 4.8 L of plasma was exchanged for the same volume of normal fresh frozen plasma. The plasma exchange, which occurred over 4 h, was aimed at replenishing coagulation factors whose synthesis has been compromised by liver failure and preventing secondary multiorgan failures, such as edema, ascites, and bleeding. After undergoing both therapies two times, his consciousness level improved. Although proteinuria resolved during therapy, hematuria did not; therefore, on day 52, he was also started on oral mizoribine at 100 mg/day. Once the patient’s blood level of mizoribine was confirmed to be normal, he was discharged on day 67. Although urine occult blood (3 +) persisted at discharge, proteinuria decreased to 0.41 g/gCr, and BUN and serum Cr levels decreased to 45.4 mg/dL and 2.22 mg/dL, respectively. Therefore, he was scheduled for fortnightly follow-up visits (Fig. 3).

## Discussion and conclusion

This case suggests the possibility that IgA nephropathy can develop, worsen, and lead to RPGN following SARS-CoV-2 vaccination. In recent case reports, IgA developed or recurred following SARS-CoV-2 vaccination.

The mechanism of action of the Pfizer-BioNTech mRNA vaccine (BNT162b2) is as follows: The vaccine, which is administered intramuscularly, combines messenger RNA (mRNA) (which expresses the particulate protrusion, the S protein, used by SARS-CoV-2 to infect cells) with reagents that transport the mRNA inside the cells [17]. An mRNA vaccine is a virus antigen template [in the case of coronavirus disease 2019 (COVID-19) mRNA vaccines, a template that produces part or all spike protein as a virus antigen] that is enveloped in a lipid membrane and transported to the target cell. The mRNA is incorporated into the cell and released into the cytoplasm, where it is translated into proteins by ribosomes. This protein is either cleaved into small portions by proteasomes or transported outside the cell by the Golgi apparatus. The remaining small portion inside the cell is expressed on the cell surface as a complex with a major histocompatibility complex class I protein and is recognized by CD8 + T cells, which induce a cell-mediated immune response. In contrast, extracellular spike proteins may be absorbed by other immune cells and cleaved by ribosomes. These portions are expressed on cell surfaces as complexes with a major histocompatibility complex class II protein and are recognized by CD4 + T cells; they promote B cells that produce antibodies specific to antigens. Additionally, lipid nanoparticle nucleoside-modified mRNAs, such as Pfizer-BioNTech and Moderna, encode the SARS-CoV-2 spike protein mediating host attachment and SARS-CoV-2 viral entry [18].

Next, we discuss the dynamics of T cells in IgA nephropathy. Although T cells in the blood of patients with IgA nephropathy are considered to be Th2-dominant [19, 20], some patients have demonstrated polarization toward Th1 [21, 22]. In studies conducted with ddY mice, the polarization toward Th1 was involved in the onset of nephritis [23]; it was hypothesized that marrow-derived Th1 cells induce nephritis, which alters the response of IgA to antigens either by abnormalities in innate immunity or by a Th2-biased background [24].

In a recent study, two doses of an mRNA vaccine activated CD4 + and CD8 + T cells [25]. This suggests that the activation of CD4 + and CD8 + T cells by mRNA vaccines may result in the exacerbation of IgA nephropathy.

In our patient, alcoholic cirrhosis of the liver inhibited the clearance of IgA from the blood, resulting in occult deposition of IgA in the glomeruli. The SARS-CoV-2 vaccine conceivably activated CD4 + and CD8 + T cells, thereby exacerbating IgA nephropathy.

In this case, steroid pulse therapy caused acute liver failure, which made monotherapy difficult; therefore, we selected low-toxicity mizoribine as an additional treatment. The use of steroid therapy and mizoribine has been reportedly effective in treating IgA nephropathy secondary to cirrhosis [26]. Therefore, if renal dysfunction worsens in the future, induction of remission using glucocorticoids is unlikely, and hemodialysis will be considered.

This case suggests that SARS-CoV-2 vaccines, particularly mRNA vaccines, may contribute to the exacerbation of IgA nephropathy. However, additional case reports and studies are warranted to consolidate this evidence.

## Data Availability

All data generated or analyzed during this study are included in this published article.

## Abbreviations

ANCA: Antineutrophil cytoplasmic antibody
BUN: Blood urea nitrogen
Cr: Creatinine
HPF: High power field
mRNA: Messenger RNA
RNA: Ribonucleic acid
RPGN: Rapidly progressive glomerulonephritis
SARS-CoV-2: Severe acute respiratory syndrome coronavirus 2
